# Transcriptome Characterization by RNA-seq Unravels the Mechanisms of Butyrate-Induced Epigenomic Regulation in Bovine Cells

**DOI:** 10.1371/journal.pone.0036940

**Published:** 2012-05-15

**Authors:** Sitao Wu, Robert W. Li, Weizhong Li, Cong-jun Li

**Affiliations:** 1 Center for Research in Biological Systems, University of California San Diego, San Diego, California, United States of America; 2 United States Department of Agriculture–Agricultural Research Service, Bovine Functional Genomics Laboratory, Beltsville, Maryland, United States of America; Peking University Health Science Center, China

## Abstract

Short-chain fatty acids (SCFAs), especially butyrate, affect cell differentiation, proliferation, and motility. Butyrate also induces cell cycle arrest and apoptosis through its inhibition of histone deacetylases (HDACs). In addition, butyrate is a potent inducer of histone hyper-acetylation in cells. Therefore, this SCFA provides an excellent *in vitro* model for studying the epigenomic regulation of gene expression induced by histone acetylation. In this study, we analyzed the differential *in vitro* expression of genes induced by butyrate in bovine epithelial cells by using deep RNA-sequencing technology (RNA-seq). The number of sequences read, ranging from 57,303,693 to 78,933,744, were generated per sample. Approximately 11,408 genes were significantly impacted by butyrate, with a false discovery rate (FDR) <0.05. The predominant cellular processes affected by butyrate included cell morphological changes, cell cycle arrest, and apoptosis. Our results provided insight into the transcriptome alterations induced by butyrate, which will undoubtedly facilitate our understanding of the molecular mechanisms underlying butyrate-induced epigenomic regulation in bovine cells.

## Introduction

Short-chain fatty acids (SCFAs), such as acetate, propionate, and butyrate, are important nutrients in ruminants. SCFAs are produced during the microbial fermentation of dietary fiber in the gastrointestinal tract and are directly absorbed at the site of production and oxidized for cell energy production and use [1]. In humans, colonic microbiota convert dietary fiber into prodigious amounts of SCFAs that benefit the human host through numerous metabolic, trophic, and chemopreventative effects [2]. The SCFA butyrate, in particular, also serves as an inhibitor of histone deacetylases (HDACs), which are critical epigenetic regulators [3], [4], [5]. Therefore, butyrate could act to reactivate epigenetically silenced genes by increasing global histone acetylation [6]. Epigenetic modifications play a key role in the regulation of gene expression, and HDAC activity contributes significantly to epigenetic modification. The HDACs are part of a transcriptional co-repressor complex that influences various tumor suppressor genes. HDACs also play significant roles in several human cancers, making HDAC inhibitors an important emerging class of chemotherapeutic agents.

Chromatin modification has evidently evolved to be a very important mechanism for the epigenetic regulation of the transcriptional status of a genome [4]. Butyrate is not only important for its nutritional impact. It also has profound impacts at the gene level, altering cell differentiation, proliferation, and motility and inducing cell cycle arrest and apoptosis [3]. The foremost biochemical change induced by butyrate and other HDAC inhibitors is the global hyper-acetylation of histones [3], [7]. Clear evidence has linked modifications in chromatin structure to cell cycle progression, DNA replication, and overall chromosome stability [8], [9]. Cultured bovine cells respond to the hyper-acetylation of histones induced by butyrate at physiological concentrations by arrest in the early G1 phase and the cessation of DNA synthesis. Butyrate at a relatively high concentration also induces apoptosis in an established bovine cell line, the Madin-Darby bovine kidney epithelial cell line (MDBK) [3]. The modulation of genome expression through chromatin structural changes by processes such as histone acetylation is considered a major genetic control mechanism.

Histone lysine acetylation has emerged as an essential regulator of genome organization and function. As a HDAC inhibitor (HDACi), butyrate is a strong inducer of the hyper-acetylation of histone in cells and provides an excellent *in vitro* model for the study of the epigenomic regulation of gene expression induced by histone acetylation. An investigation of the global gene expression profiles of MDBK cells and their regulation by sodium butyrate has recently been conducted using a high-density oligonucleotide microarray [10]. The profound changes observed in gene expression in bovine cells following butyrate treatment demonstrate the pleiotropic effects of histone acetylation [5]. As nutrition research shifts from epidemiology and physiology to the study of molecular interactions with the genome and the elucidation of these less-obvious nutritional effects, a detailed knowledge of changes in gene expression becomes necessary as a basis for understanding these molecular mechanisms.

In the present study, we report our findings on the function and pathways induced by butyrate in MDBK cells. We used deep RNA sequencing to provide a significant amount of novel gene information for bovine cell transcription, which can then be used for further transcriptomic studies or to gain a deeper understanding of the bovine genome and transcriptome. This study also provides a significant amount of information for the epigenetic regulation induced by butyrate. Our data show that butyrate-induced histone acetylation results in subsequent changes in the accessibility of the DNA to transcription activities. Transcriptomic characterization using deep RNA sequencing facilitates the identification of the potential mechanisms underlying gene expression and the epigenomic regulation of cellular functions induced by butyrate.

## Results

### Butyrate treatment induces changes in cell morphology and cell cycle arrest

**Figure 1:**
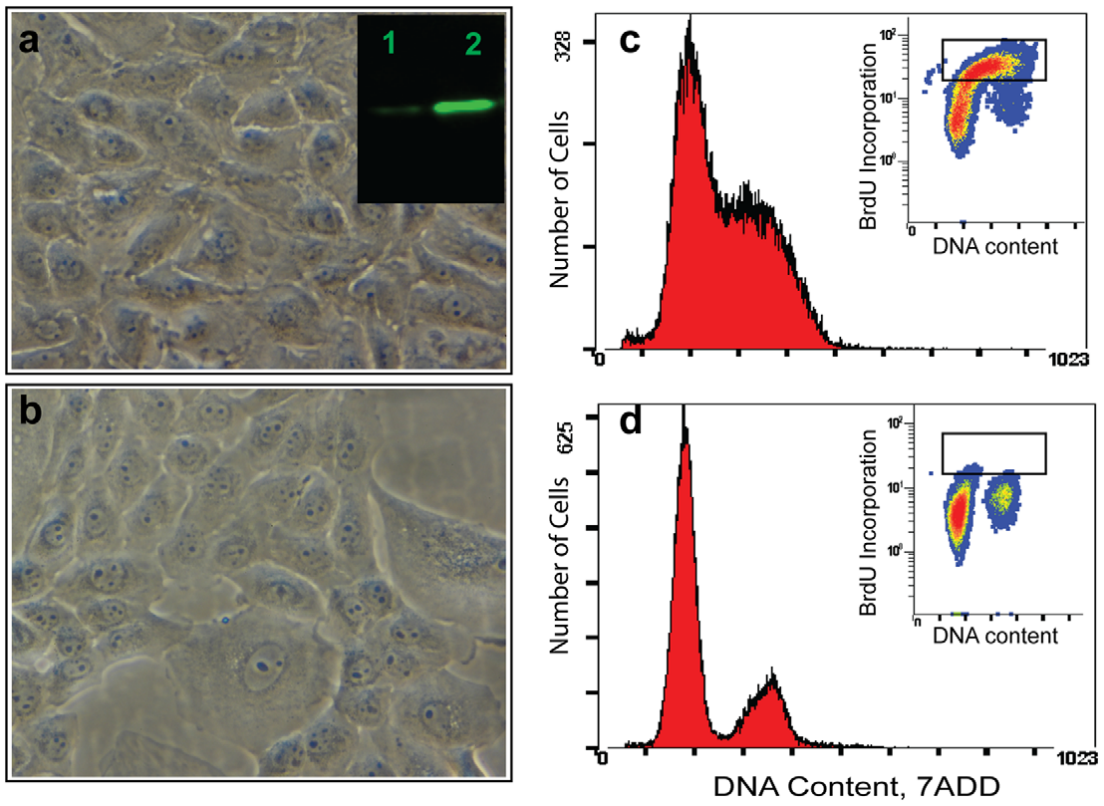
Butyrate induces significant biological effects in cultured MDBK cells. a): normal cells; b): cells treated with10 mM butyrate for 24 hrs, showing morphological changes including large vacuoles, ragged membranes, lack of distinct intracellular organelles, and increasing spaces between cells. Insert in a) comparison of histone H3 acetylation of normal cells (1) and histone acetylation in butyrate-treated cells (2). c and d: Cell population profiles determined by flow cytometry. c); normal cells and d) cells treated with butyrate. Inserts: BrdU labeling show butyrate blocked the DNA synthesis after 24 hr treatment. Cells were first pulse labeled with BrdU for 30 min. Collected cells were first stained with diluted fluorescent (Fluorescent isothiocyanate, FITC) anti-BrdU antibody and then stained with DNA marker (7-ADD). The fluorescent signal generated by FITC was acquired in a logarithmic mode, and fluorescent signal from the DNA-content marker 7-ADD was normally acquired in the linear signal amplification mode. Cells were separated into three clusters by double staining analysis. Butyrate treatment eliminates cells in S phase (in rectangle box).

We previously reported that butyrate induces cell cycle arrest in MDBK cells. In preparation for deep RNA sequencing, we first endeavored to confirm that the butyrate induced cell cycle arrest. When cells were treated with 10 mM butyrate for 24 hours, cell morphology became distorted. Cells with large vacuoles, with ragged membranes, lacking distinct intracellular organelles, and having increased spaces between cells were readily visible and recurrent. Flow cytometry analysis of the cell population profiles for DNA content and BrdU labeling also confirmed that the cells were arrested at the G1 and G1/S boundary. The incorporation of the BrdU label suggested that DNA synthesis was blocked by butyrate treatment. Western blotting also confirmed that butyrate induced the hyper-acetylation of H3 (Figure 1).

### RNA-seq provided a comprehensive view of the bovine cell transcriptome

In total, 57,303,693 to 78,933,744 sequence reads were generated per sample (Table 1), and 24,526 genes had at last one sequence hit in at least one sample. Of these, 16,212 genes were shared by all samples and can be considered to be the core transcriptome of the bovine epithelial cell. A mean value of 19,477±155 (mean±SD) genes was detected in the butyrate-treated group, while 17,626±125 (mean±SD) genes were detected in the control group. Table 2 summarizes the alignment results. Among these genes, 11,408 genes showed a significant differential expressed at a strict false discovery rate (FDR) <0.05 (Table S1).

**Table 1:** Summary of RNA-Seq coverage data.

Sample ID	Yield (Mbases)	# Reads	% of raw clusters per lane	% Perfect Index Reads	% One Mismatch Reads (Index)	% of > = Q30 Bases (PF)	Mean Quality Score (PF)
BT1	3,371	67,423,659	39.48	85.92	14.08	89.37	35.44
BT2	3,365	67,298,291	39.98	80.36	19.64	89.23	35.42
BT3	2,865	57,303,693	33.57	81.88	18.12	89.45	35.43
BT4	3,947	78,933,744	44.68	88.33	11.67	89.56	35.54
C1	3,918	78,367,052	36.59	92.6	7.4	90.83	35.87
C2	2,910	58,207,130	43.22	92.63	7.37	87.2	34.75
C3	3,551	71,026,638	43.7	60.27	39.73	88.46	35.12
C4	3,083	61,656,681	47.79	95.99	4.01	87.17	34.83

BT; butyrate treated; C: Control.

**Table 2:** Summary of the bovine transcriptome in MDBK cells (Genes with at least one hit).

Sample replicate	Butyrate -treated	Control
1	19207	17804
2	19248	17523
3	19282	17615
4	19550	17560
Mean	19477	17626
SD	155	125

P-value  = 0.0000026

Previous gene expression profiling in MDBK cells and the induction of histone acetylation by butyrate has been analyzed by using bovine oligonucleotide microarray. In this previous study, 30 genes representing different expression levels and functional classes were selected and validated by real-time RT-PCR [5]. We were able to confirm over 70% of the differentially regulated genes that were identified by the microarray experiment using RNA-seq. However, RNA-seq allowed us to identify a significantly greater numbers of genes that were induced by butyrate, but which had not been previously associated with the biological effects of butyrate. Transcriptome characterization by RNA-seq also identified 587 genes that were uniquely expressed in butyrate-treated cells, but had not been previously detected by a microarray experiment in cells given similar treatments [5].

### Functional annotation of differentially expressed genes induced by butyrate

The biological relevance of butyrate-induced gene expression in bovine cells was explored by the Gene Ontology (GO) classification. Table 3 lists 65 GO terms that were significantly perturbed by butyrate treatment. The most-represented biological processes and molecular functions, sorted by statistical significance in both terms of p–value and FDR, included nucleic acid metabolic process, DNA metabolic processes, the regulation of the cell cycle, and DNA replication.

**Table 3:** Butyrate treatment induces changes in major GO processing.

GO ID	Description	Ratio in Study	Ratio in Population	p-value	FDR
GO:0090304	Nucleic acid metabolic process	1226/11408	1618/16591	5.87E-10	0.000
GO:0036094	Small molecule binding	1489/11408	1973/16591	5.89E-10	0.000
GO:0034641	Cellular nitrogen compound metabolic process	1798/11408	2400/16591	6.68E-10	0.000
GO:0006139	Nucleobase-containing compound metabolic process	1612/11408	2146/16591	6.71E-10	0.000
GO:0006807	Nitrogen compound metabolic process	1837/11408	2469/16591	6.76E-10	0.000
GO:0044260	Cellular macromolecule metabolic process	2593/11408	3496/16591	7.02E-10	0.000
GO:0000166	Nucleotide binding	1402/11408	1862/16591	7.02E-10	0.000
GO:0044446	Intracellular organelle part	2447/11408	3252/16591	7.03E-10	0.000
GO:0005634	Nucleus	2303/11408	3089/16591	7.32E-10	0.000
GO:0044444	Cytoplasmic part	2764/11408	3742/16591	7.45E-10	0.000
GO:0005737	Cytoplasm	2097/11408	2778/16591	7.64E-10	0.000
GO:0044237	Cellular metabolic process	3632/11408	4917/16591	7.89E-10	0.000
GO:0044422	Organelle part	2482/11408	3308/16591	7.92E-10	0.000
GO:0043170	Macromolecule metabolic process	2859/11408	3905/16591	8.05E-10	0.000
GO:0044238	Primary metabolic process	3647/11408	4986/16591	8.21E-10	0.000
GO:0003674	Molecular_function	8856/11408	12490/16591	8.32E-10	0.000
GO:0043227	Membrane-bounded organelle	3833/11408	5154/16591	8.34E-10	0.000
GO:0043229	Intracellular organelle	4370/11408	5881/16591	8.78E-10	0.000
GO:0044464	Cell part	7397/11408	10297/16591	8.81E-10	0.000
GO:0003824	Catalytic activity	3290/11408	4535/16591	8.82E-10	0.000
GO:0043226	Organelle	4376/11408	5887/16591	8.85E-10	0.000
GO:0005575	Cellular_component	7714/11408	10774/16591	8.89E-10	0.000
GO:0005515	Protein binding	5263/11408	7294/16591	8.94E-10	0.000
GO:0008150	Biological_process	7442/11408	10453/16591	9.01E-10	0.000
GO:0043231	Intracellular membrane-bounded organelle	3828/11408	5149/16591	9.26E-10	0.000
GO:0005488	Binding	7434/11408	10421/16591	9.39E-10	0.000
GO:0008152	Metabolic process	4284/11408	5856/16591	9.42E-10	0.000
GO:0009987	Cellular process	5831/11408	8120/16591	9.53E-10	0.000
GO:0044424	Intracellular part	5586/11408	7576/16591	1.02E-09	0.000
GO:0044428	Nuclear part	1045/11408	1375/16591	1.14E-09	0.000
GO:0050789	Regulation of biological process	3605/11408	5012/16591	6.52E-09	0.000
GO:0065007	Biological regulation	3746/11408	5215/16591	6.90E-09	0.000
GO:0043234	Protein complex	1507/11408	2030/16591	9.92E-09	0.000
GO:0006259	DNA metabolic process	341/11408	423/16591	3.66E-08	0.000
GO:0035639	Purine ribonucleoside triphosphate binding	1153/11408	1540/16591	3.96E-08	0.000
GO:0032555	Purine ribonucleotide binding	1160/11408	1551/16591	5.16E-08	0.000
GO:0032553	Ribonucleotide binding	1160/11408	1551/16591	5.16E-08	0.000
GO:0050794	Regulation of cellular process	3389/11408	4718/16591	6.51E-08	0.000
GO:0017076	Purine nucleotide binding	1162/11408	1556/16591	8.73E-08	0.000
GO:0048518	Positive regulation of biological process	1395/11408	1885/16591	1.27E-07	0.000
GO:0031090	Organelle membrane	656/11408	855/16591	1.59E-07	0.000
GO:0009058	Biosynthetic process	1443/11408	1954/16591	1.72E-07	0.000
GO:0044249	Cellular biosynthetic process	1379/11408	1864/16591	1.95E-07	0.000
GO:0048523	Negative regulation of cellular process	1136/11408	1525/16591	2.79E-07	0.000
GO:0048522	Positive regulation of cellular process	1262/11408	1704/16591	4.98E-07	0.000
GO:0048519	Negative regulation of biological process	1222/11408	1649/16591	6.09E-07	0.000
GO:0033554	Cellular response to stress	384/11408	488/16591	8.35E-07	0.000
GO:0080090	Regulation of primary metabolic process	1874/11408	2574/16591	1.17E-06	0.002
GO:0005730	Nucleolus	269/11408	334/16591	1.20E-06	0.002
GO:0051726	Regulation of cell cycle	301/11408	377/16591	1.21E-06	0.002
GO:0051236	Establishment of RNA localization	51/11408	53/16591	1.53E-06	0.002
GO:0050658	RNA transport	51/11408	53/16591	1.53E-06	0.002
GO:0050657	Nucleic acid transport	51/11408	53/16591	1.53E-06	0.002
GO:0006950	Response to stress	689/11408	909/16591	1.65E-06	0.002
GO:0005524	ATP binding	913/11408	1222/16591	2.36E-06	0.002
GO:0032559	Adenyl ribonucleotide binding	917/11408	1228/16591	2.49E-06	0.002
GO:0005815	Microtubule organizing center	229/11408	282/16591	2.65E-06	0.002
GO:0005813	Centrosome	197/11408	240/16591	3.14E-06	0.002
GO:0051028	mRNA transport	42/11408	43/16591	3.16E-06	0.002
GO:0031323	Regulation of cellular metabolic process	1886/11408	2597/16591	3.19E-06	0.002
GO:0015931	Nucleobase-containing compound transport	58/11408	62/16591	3.20E-06	0.002
GO:0019222	Regulation of metabolic process	2104/11408	2907/16591	3.30E-06	0.002
GO:0006260	DNA replication	89/11408	100/16591	3.52E-06	0.002
GO:0031570	DNA integrity checkpoint	48/11408	50/16591	3.82E-06	0.003
GO:0030554	Adenyl nucleotide binding	919/11408	1233/16591	4.15E-06	0.003

### Global function and pathway analyses identified the mechanism of butyrate-induced cell cycle arrest

The functional category and pathway analysis of differentially expressed genes in cells treated with butyrate were explored using the IPA (Ingenuity Pathways Analysis, Ingenuity® Systems, www.ingenuity.com) Knowledge Base. Of the 24,525 genes in the data set, 13,885 genes were mapped, and 10,637 genes were not mapped in the database. These genes were uploaded for IPA. Among the 13,885 mapped genes, 8,862 genes were identified with matched gene symbols and were used in pathway analysis. Of these, 5,542 genes were significantly up-regulated, while 3,320 genes were significantly down-regulated by butyrate. In comparison, the earlier microarray reports [10] identified only 371 genes (285 genes down- and 86 up-regulated genes) for the IPA analysis.

Functional analysis identified the biological functions and/or diseases that were most significantly enriched in the dataset. When the functional category analysis of the genes was performed, genes from the datasets that were associated with biological functions and/or diseases in the Ingenuity Pathways Knowledge Base were considered for analysis. Fischer's exact test was used to calculate the *P* values. The top five molecular and cellular functions, as determined by *P*-value, are listed in Table 4. These five functional categories may represent the mechanisms underlying the essential biological effects of butyrate treatment, including cell morphology changes, cell cycle arrest, and apoptosis. The number of genes defined in each function category was greatly extended by RNA-seq to include 2,257 genes involved in cell death and 2,322 genes involved in cellular growth and proliferation (Table 4).

**Table 4:** Summary of top molecular and cellular function regulated by butyrate.

Function Annotation	p-Value	Number of Genes
Cell Death	7.27E-49	2257
Gene Expression	9.41E-48	1544
Cellular Growth and Proliferation	4.88E-39	1420
Cellular Assembly and Organization	1.49E-28	1164
Cellular Function and Maintenance	1.49E-28	1565
Cell Cycle	5.86E-28	1117
Molecular Transport	1.54E-24	1320
Post-Translational Modification	4.02E-23	1074
Cellular Movement	9.17E-21	1318
Cellular Development	2.76E-19	1701
Cell Signaling	3.69E-16	692
Carbohydrate Metabolism	1.01E-14	572
DNA Replication, Recombination, and Repair	1.71E-14	718
Cellular Compromise	4.21E-14	145

**Figure 2:**
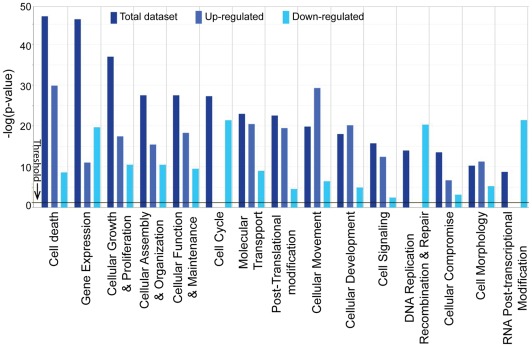
Global functional analysis. Comparison of three datasets (up-, down-regulated gene datasets and combined dataset. Datasets were analyzed by the Ingenuity Pathways Analysis software (Ingenuity® Systems, www.ingenuity.com). The significance value associated with a function in Global Analysis is a measure for how likely it is that genes from the dataset file under investigation participate in that function. The significance is expressed as a *p*-value, which is calculated using the **right-tailed Fisher's Exact Test**.

We illustrated the functional changes induced by butyrate treatment by separately comparing the functional categories that were up- or down-regulated. Figure 2 shows the top fifteen functional categories that were significantly enriched in either up- or down-regulated genes. Cell cycle; DNA replication, recombination, and repair; and RNA post-transcriptional modification were among the functional categories that were significantly impaired by butyrate. In contrast, cell death, cellular growth and proliferation, molecular transport, and cellular signaling categories were enhanced by butyrate.

**Figure 3:**
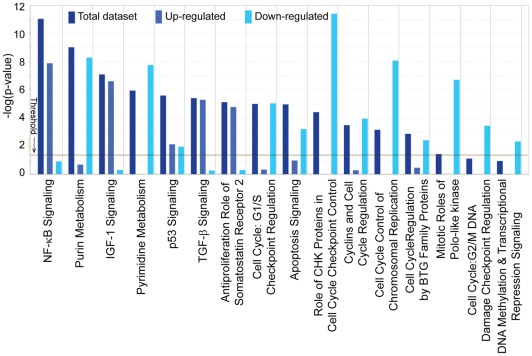
Global Canonical Pathway analysis: Comparison of three datasets (**up-, down-regulated gene datasets, and a combined dataset.** Datasets were analyzed by the Ingenuity Pathways Analysis software (Ingenuity® Systems, www.ingenuity.com). The significance is expressed as a *p*-value, which is calculated using the **right-tailed Fisher's Exact Test**.

Four canonical pathways (Cell cycle G2/M DNA damage checkpoints, purine metabolism, pyrimidine metabolism, and G1/S checkpoint regulation) previously identified by the microarray experiment were also confirmed by the RNA-seq analysis. In addition, many other pathways were significantly impacted by butyrate treatment, including those directly related to cell cycle regulation, DNA replication, and cell cycle control of chromosomal replication; these finding were consistent with the observed phenotypic changes in cell cycle arrest and the blockage of DNA synthesis induced by butyrate. Signaling pathways, including NF-κB, IGF-1, p53, TGF-β, and apoptosis signaling, were also significantly induced by butyrate (Figure 3 and Table S2).

### Butyrate induced extensive deregulation of genes related to cell cycle progression

IPA analysis identified 1,117 genes associated with cell cycle progression that were differentially regulated by butyrate (p-value: up to 5.86E^−28^) (Table 4). These genes were involved in various checkpoint pathways and selected examples of these pathways were analyzed in further detail. A complete list of pathways is presented in the supplementary material (Table S3).

**Figure 4:**
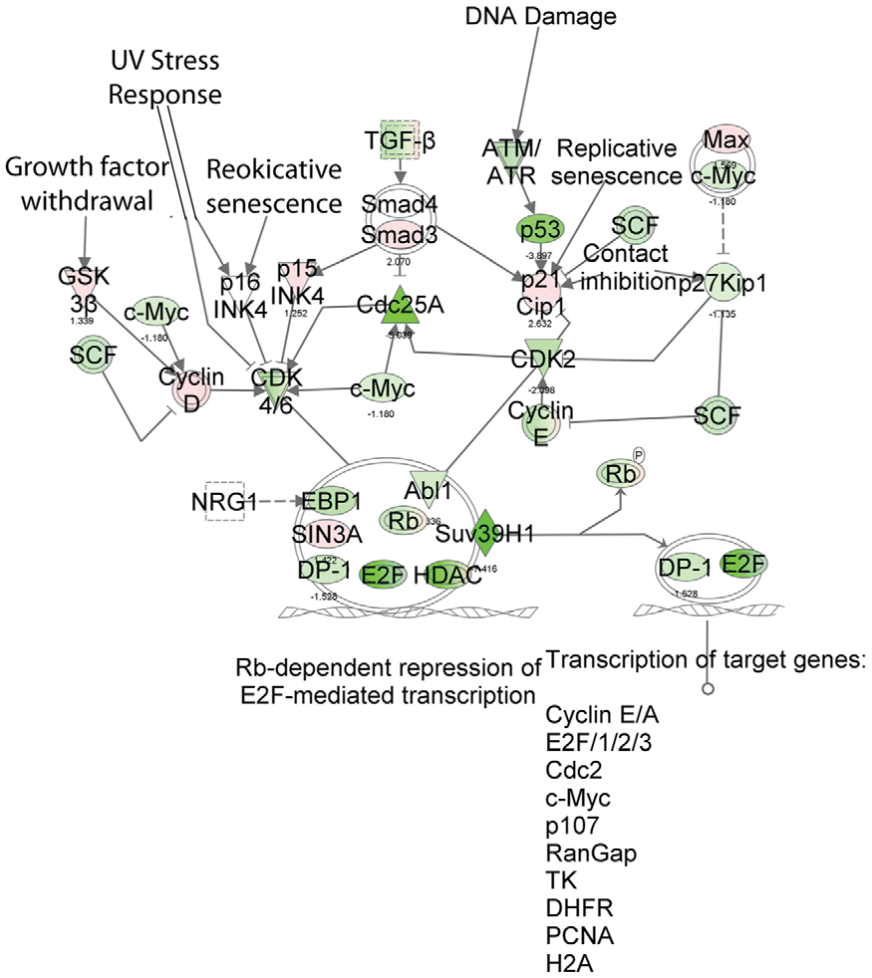
The biologically relevant pathways: Regulation of the cell cycle: The G1/S checkpoint control. The dataset was analyzed by the Ingenuity Pathways Analysis software (Ingenuity® Systems, www.ingenuity.com). The color indicates the expression level of the genes (red indicating up-regulated genes and green indicating down-regulated genes).

Regulation of the cell cycle: The G1/S checkpoint control is vital for normal cell division. Deregulation of the expression of checkpoint proteins can lead to apoptosis or tumorigenesis. This pathway highlights the key components of G1/S checkpoint regulation. Our data indicated that the G1/S checkpoint regulation pathway is one of the significantly down-regulated canonical pathways, as 46 of the 61 genes in the pathway, (ABL1, ATM, ATR, BTRC, CCND1, CCND2, CCND3, CCNE1, CCNE2, CDC25A, CDK2, CDK4, CDK6, CDKN1A, CDKN1B, CDKN2B, E2F1, E2F2, E2F3, E2F4, E2F6, GSK3B, HDAC1, HDAC2, HDAC3, HDAC4, HDAC5, HDAC6, HDAC7, HDAC9, HDAC10, HDAC11, MAX, MYC, PA2G4, RB1, RBL1, RBL2, SIN3A, SKP2, SMAD3, SUV39H1, TFDP1, TGFB2, TGFB3, TP53) were deregulated by butyrate treatment. Among the 46 genes, 27 were down-regulated (Figure 4). It is very interesting to note that HDACs were among these deregulated genes; for example, HCACs 1, 4, 7, 9, and 10 were down-regulated, while HDACs 2, 3, 5, 6, and 11 were significantly up-regulated by butyrate.

**Figure 5:**
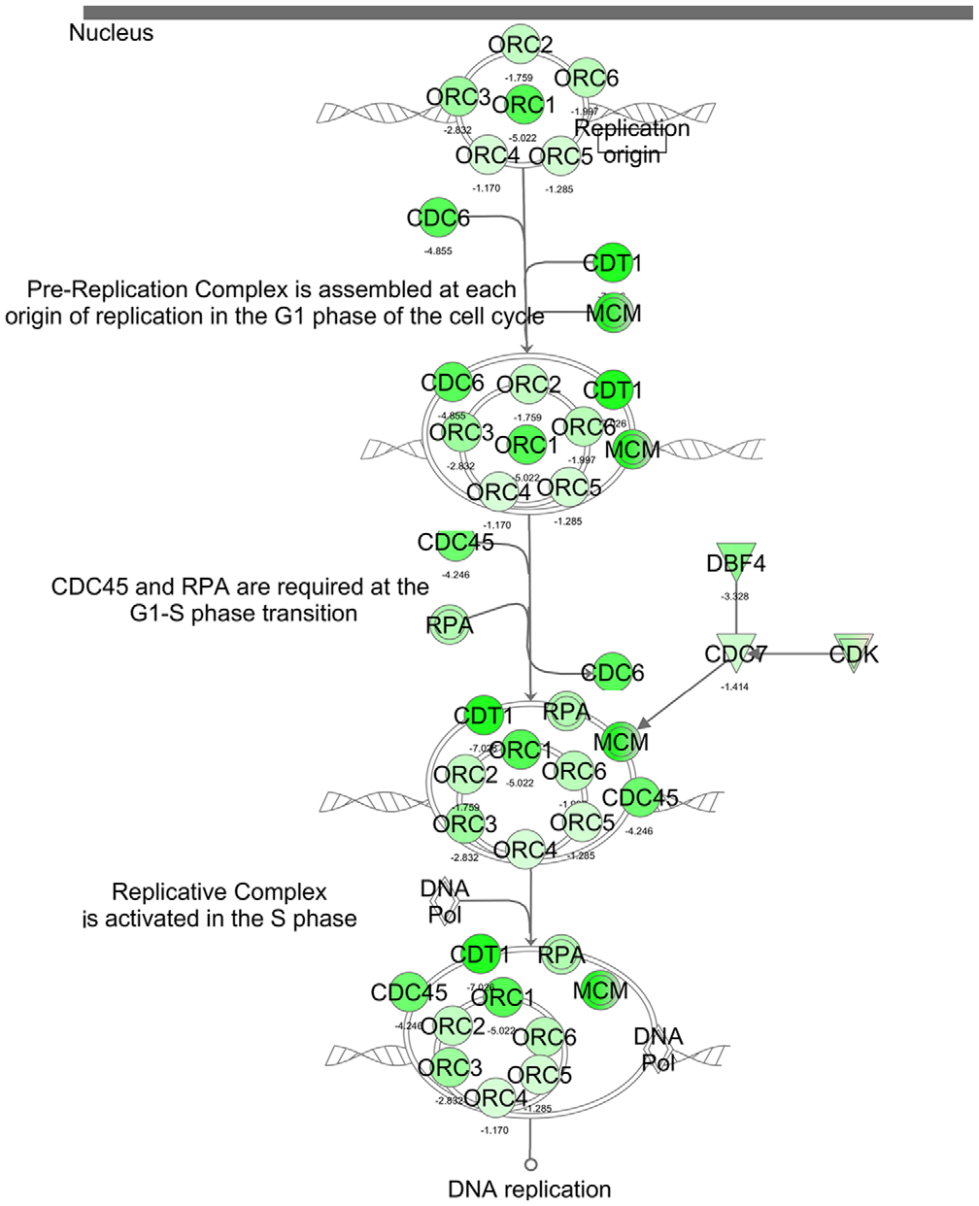
The biological relevant pathways: Cell Cycle Control of Chromosomal Replication. Data set was analyzed by the Ingenuity Pathways Analysis software (Ingenuity® Systems, www.ingenuity.com). The color indicates the expression level of the genes (red indicating up-regulated genes and green indicating down-regulated genes).

Regulation of DNA replication: cell cycle control of chromosomal replication is another canonical pathway closely related to cell cycle progression. The top functions of these pathways included DNA replication, recombination, and repair; cell cycle regulation; cellular assembly; and cellular organization. The stable propagation of genetic information requires that the entire genome of an organism be faithfully replicated only once in each cell cycle. Therefore, chromosomal DNA replication in eukaryotic cells entails a series of complex events that includes the recognition of origins, the firing of replication origins, the loading of DNA polymerases onto the origins, and the elongation of newly synthesized DNA. The initiation of DNA replication takes place only at specific loci on the chromosomal DNA, which are termed replication origins. The Origin Recognition Complex (ORC) includes six components (ORC1 to ORC6), which are specifically associated with replication origin throughout the cell cycle. ORC serves as a hallmark of the origins and is highly conserved. ORC1 is the largest subunit of the origin recognition complex and the association of ORC1 with chromatin appears to be the rate-limiting step in the assembly of a functional pre-replication complex [11]. Our data revealed that 23 genes from a total of 30 genes involved in this pathway were regulated by butyrate. These genes (such as CDC45, CDC6, CDC7, CDK, CDT1, CHEK2, DBF4, DNA Polymerase, MCM, ORC, ORC-CDC45-CDT1-MCM-RPA, ORC1, ORC2, ORC3, ORC4, ORC5, ORC6, which are the important components for the formation of the pre-replication complex, as well as RC and RPA) were all significantly down-regulated (Figure 5).

**Figure 6:**
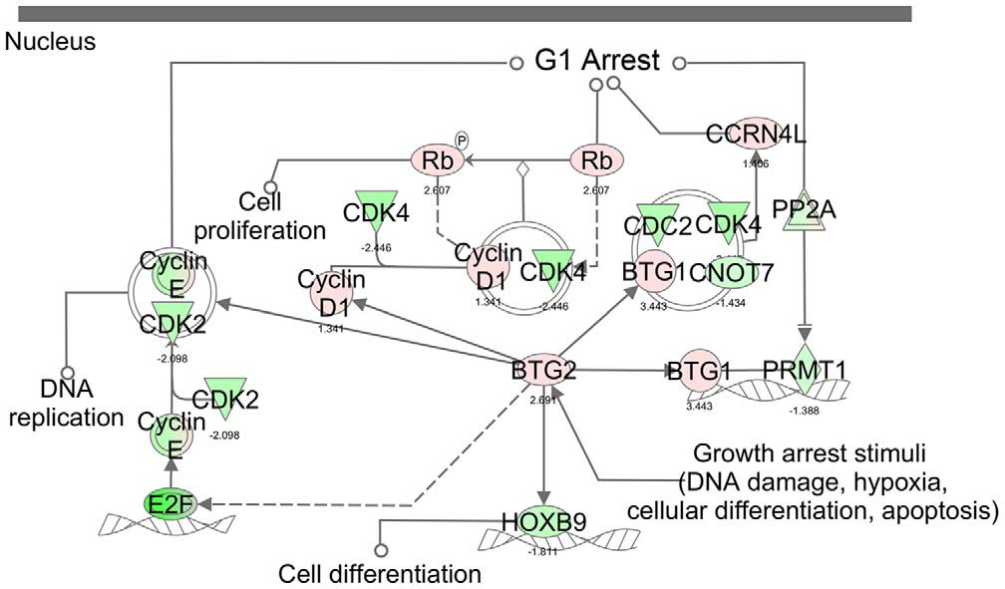
The biological relevant pathways: Cell cycle regulation by BTG proteins. The dataset was analyzed by the Ingenuity Pathways Analysis software (Ingenuity® Systems, www.ingenuity.com). The color indicates the expression level of the genes (red indicating up-regulated genes and green indicating down-regulated genes).

The canonical pathway of cell cycle regulation by BTG proteins may also play an important role in butyrate-induced cell cycle arrest. As shown in Figure 6, both BTG1 and BTG2 were up-regulated by butyrate treatment. BTG1 expression reaches a maximum in the G0/G1 phases of the cell cycle and then begins to undergo down-regulation as cells progress through G1. BTG1 negatively regulates cell proliferation [12]. BTG2 proteins are anti-proliferation proteins involved in cell cycle regulation, growth arrest, and differentiation. The activation of BTGs may lead to the down-regulation of the cyclin E/CDK2 complex and other members of the cyclin family that are essential for the progression of the cell cycle from G1 to the S phase and that are responsible for the regulation of cyclin-dependent kinases. All of these differentially expressed genes and their functions are in agreement with our results and with the observed biological effects of butyrate.

Our data also demonstrated that cytokinesis was significantly down-regulated, with a p-value of 5.22E^−09^and a Z-score of −2.781 (Table 5). A total of 48 genes in this pathway were down-regulated, including Aurora kinases A, B and C. The KIF (kinesin superfamily of microtubule-associated motors) members, such as KIF 4A, C1, 20A, 23, were also significantly down-regulated (from −4.0 to −7.6 fold). These findings confirm the earlier microarray results that showed that butyrate induced changes in the expression of genes related to cytokinesis [5].

**Table 5:** Butyrate down-regulated the major functions involved in cell cycle regulation.

Functions Annotation	p-Value		Predicted Activation State	Regulation z-score	Number of genes
Cell Cycle	4.73E-28– 9.26E-05	Decreased			1117
Cell cycle progression	4.73E-28			0.190	718
Interphase	1.86E-25	Decreased		−2.439	484
G1 phase	1.71E-15	Decreased		−2.524	278
Cytokinesis	5.22E-09	Decreased		−2.781	90
Interphase of fibroblasts	4.26E-06	Decreased		−2.615	48
Interphase of connective tissue cells	4.99E-06	Decreased		−2.474	52

Transcription factors: Transcription factors are a group of proteins that bind to specific DNA sequences and control the transcription of genetic information from DNA to mRNA [13]. Transcription factors either promote (as activators) or block (as repressors) the recruitment of RNA polymerase to specific genes. Table 6 lists the major transcription factors identified by RNA-seq and IPA analysis that were involved in the regulatory effect of butyrate. Only the genes with a predicted activation state, either activated or inhibited, are listed.

**Table 6:** Major transcription regulators with defined activities regulated by butyrate treatment.

Transcription Regulator	Predicted Activation State	Regulation z-score	p-value of overlap
TP53	Activated	3.288	2.91E-28
KDM5B	Activated	3.232	3.43E-05
IRF3	Activated	3.145	1.57E-01
Creb	Activated	2.794	1.71E-02
MXI1	Activated	2.693	2.03E-02
OTX2	Activated	2.678	2.08E-01
EZH2	Activated	2.557	3.59E-01
IRF1	Activated	2.500	3.60E-03
NR3C2	Activated	2.482	7.11E-02
ELF4	Activated	2.427	1.32E-01
CBX4	Activated	2.383	2.43E-01
MYOG	Activated	2.291	1.07E-01
MSX2	Activated	2.211	7.31E-02
HTT	Activated	2.168	2.01E-04
CEBPA	Activated	2.166	9.32E-07
Rb	Activated	2.158	1.88E-03
POU4F1	Activated	2.065	5.82E-03
JUNB	Activated	2.047	1.72E-01
NR3C1	Activated	2.024	2.71E-17
STAT5A	Activated	2.023	1.47E-03
N-cor	Inhibited	−2.071	3.50E-01
MEF2A	Inhibited	−2.086	3.74E-01
SP3	Inhibited	−2.311	2.38E-03
Ctbp	Inhibited	−2.368	2.03E-02
KAT5	Inhibited	−2.442	6.45E-02
IRF9	Inhibited	−2.447	3.83E-01
HSF2	Inhibited	−2.481	8.28E-02
E2F3	Inhibited	−2.492	4.99E-04
Hdac	Inhibited	−2.789	1.77E-02
FOXM1	Inhibited	−3.064	4.61E-03
SREBF2	Inhibited	−3.150	4.67E-02
FLI1	Inhibited	−3.592	1.92E-02
MYC	Inhibited	−3.759	1.15E-11
SIRT2	Inhibited	−3.987	7.01E-03
XBP1	Inhibited	−4.077	6.15E-11
HSF1	Inhibited	−4.744	2.03E-03

TP53, one of the most important transcription factors, was found in the center of a down-regulated network in the microarray profiling of butyrate-induced regulation. However, microarrays failed to detect changes in TP53 gene expression. In the present experiment with RNA-seq, TP53 was clearly down-regulated by butyrate (∼4 fold). TP53 targets 518 genes in the entire dataset of differentially expressed genes induced by butyrate (Table S4). In addition to TP53, butyrate also induced the expression of TP53BP1, TP53BP2, TP53I13, TP53INP1 (tumor protein p53 inducible nuclear protein 1), and TP53I11. TP53INP1 was up-regulated (2.5 fold), while functionally-associated gene TP73 was up-regulated almost 24-fold. All of these changes in gene expression suggest a cell-cycle regulation network that may enhance cell cycle arrest.

The expression of non-coding RNAs (ncRNA) was disrupted by butyrate treatment: Deep RNA-seq also reveals a significant amount of information regarding ncRNA. There are 24 ncRNAs that are differentially expressed due to the butyrate treatment. Those ncRNAs belong to different types of ncRNAs, including snoRNA (small nucleolar RNA), snRNA (splicesomal RNA), and some miscRNAs (Table 7). Particularly, the expression of 10 snoRNAs (5 down-regulated and 5 up-regulated) was found to be disrupted by the butyrate treatment. snoRNAs are intermediate-sized ncRNAs (60–300 bp). They are components of small nucleolar ribonucleoproteins (snoRNPs), which are complexes that are responsible for the modification and processing of ribosomal RNA [14]. More importantly, a large proportion of snoRNAs have been found to be further processed into smaller molecules, such as microRNAs (miRNAs) [15]. Surprisingly, only one miRNA with differential expression was detected. We suspect that the RNA purification protocols may exclude small RNAs. It is certainly interesting to follow-up this finding to look into the functionality of the disruption of the expression of ncRNA induced by butyrate.

**Table 7:** Butyrate-induced disruption in non-coding RNA expression.

ENSEMBL_ID	Gene Biotype	Fold (BT/CT)	Description	locus	p_value	q_value
ENSBTAG00000044614	snoRNA	0.18	Small Cajal body specific RNA 23	6:109784641–109826922	1.01E-02	0.0153
ENSBTAG00000045102	snoRNA	0.34	Small nucleolar RNA U89	5:103853321–103857548	1.07E-04	0.0002
ENSBTAG00000042757	snoRNA	0.38	Small nucleolar RNA SNORA20	9:97466404–97492748	1.56E-08	0.0000
ENSBTAG00000043000	snoRNA	0.38	Small nucleolar RNA SNORA1	29:1066221–1066355	1.83E-02	0.0270
ENSBTAG00000042183	snoRNA	0.41	Small nucleolar RNA SNORA20	4:59551832–59551962	8.26E-05	0.0002
ENSBTAG00000047875	miRNA	0.67	Novel	19:49330953–49337523	2.79E-02	0.0402
ENSBTAG00000044453	misc_RNA	1.52	7SK RNA	4:113212666–113212975	1.28E-02	0.0192
ENSBTAG00000045419	misc_RNA	1.53	7SK RNA	6:27777684–27778005	1.31E-05	0.0000
ENSBTAG00000044427	misc_RNA	1.59	Nuclear RNase P	10:26814255–26814588	3.24E-02	0.0464
ENSBTAG00000043171	misc_RNA	1.68	7SK RNA	8:18781961–18782266	1.32E-04	0.0002
ENSBTAG00000045128	misc_RNA	1.68	7SK RNA	1:14608518–14608853	2.94E-02	0.0422
ENSBTAG00000045530	snoRNA	1.73	Novel	3:34395250–34395683	3.15E-03	0.0051
ENSBTAG00000043250	misc_RNA	1.79	7SK RNA	23:24977641–24977972	3.92E-03	0.0063
ENSBTAG00000046888	misc_RNA	2.06	Novel	6:31669691–31669966	2.77E-03	0.0045
ENSBTAG00000047075	misc_RNA	2.06	Novel	19:58435867–58436204	2.78E-02	0.0401
ENSBTAG00000044659	snoRNA	2.25	Small Cajal body specific RNA 13	21:61984641–61984915	3.23E-02	0.0462
ENSBTAG00000046063	snoRNA	3.05	Novel	19:47441443–47441573	1.39E-02	0.0208
ENSBTAG00000042354	snoRNA	3.13	SNORA3/SNORA45 family	15:44469326–44472127	1.40E-02	0.0209
ENSBTAG00000048120	misc_RNA	3.80	Novel	3:34060768–34060962	1.08E-03	0.0018
ENSBTAG00000029640	snRNA	4.65	U1 spliceosomal RNA	18:14877691–14877845	2.71E-02	0.0391
ENSBTAG00000037013	snRNA	5.80	U1 spliceosomal RNA	21:45387108–45387272	2.23E-02	0.0325
ENSBTAG00000043738	misc_RNA	6.68	7SK RNA	15:43689412–43689680	2.43E-02	0.0353
ENSBTAG00000046209	snRNA	18.19	Novel	17:20132500–20132665	1.18E-03	0.0020
ENSBTAG00000042191	snoRNA	>1000	Small nucleolar RNA U2-19	13:81838598–81838678	2.81E-02	0.0404876*

* No detectable in control samples.

## Discussion

During the last few years, several publications have reported the use of HDAC inhibitors to study histone acetylation and gene regulation. An important question to be addressed by the study of histone modification is how modifications affect not only chromatin dynamics but also various processes (e.g., DNA replication, RNA transcription) along the DNA-template. These processes can be influenced by a number of post-translational modifications of histones, including acetylation, methylation, phosphorylation, and ubiquitination. These modifications may not act alone, but in concert and in a context-dependent manner to facilitate or repress chromatin-mediated processes [6].

Our previous studies [3], [5], [10], [16] revealed that VFAs, especially butyrate, participate in metabolism, both as nutrients and as regulators of histone modification, thereby regulating the ‘epigenomic code.’ These findings implicate histone modifications induced by butyrate as determinants of bovine phenotype and in bovine ruminal development.

Epigenomics is an emerging area of scientific investigation that is confirming the complexity of the mechanisms used to determine the how, when, and where of gene expression in order to ensure the normal development, health, and homeostasis of the animal. The recently completed profiling of global gene expression used a high-density oligonucleotide microarray [5], [10] to identify 450 genes in bovine kidney epithelial cells that were significantly regulated by sodium butyrate at a very stringent false discovery rate (FDR) of 0%. The functional category and pathway analyses of the microarray data revealed that four canonical pathways (cell cycles: G2/M DNA damage checkpoint, pyrimidine metabolism, G1/S checkpoint regulation, and purine metabolism) were significantly perturbed. The biologically relevant networks and pathways of these genes were also identified, including genes such as *IGF2, TGFB1, TP53, E2F4,* and *CDC2*, which were established as central to these networks. However, because they are restricted to probes designed to target the genes in a given species' genome, hybridization-based microarray technologies offer a limited ability to fully catalogue and quantify the diverse RNA molecules that are expressed from genomes over a wide range of levels [17], and they often fail to capture the full catalogue of transcripts and their variations.

The development of the next-generation sequencing (NGS) has provided novel tools for expression profiling and genome analysis [17,18,19]. As a vital step towards a comprehensive understanding of the molecular mechanism of butyrate-induced acetylation, as well as its biological effects, the present study was designed to utilize next-generation sequencing technology in order to provide a more complete characterization of the RNA transcripts of MDBK cells. This study also focused on the comparison between the control group (without butyrate treatment) and the cells treated with 10 mM butyrate for 24 hours. With technical replicates (four lanes for controls and four lanes for butyrate-treated samples), the samples were deep-sequenced, with an average of more than 67 million reads per sample, and the results were used to estimate the differences induced by butyrate treatment. Therefore, our results show a very reliable and detailed profiling of the changes in gene expression induced by butyrate.

This study has generated comprehensive information on an experimental system that can be used in many functional genomics studies of bovine cells. To the best of our knowledge, this is the first study that has used NGS and IPA to identify the influences of butyrate on transcriptomic characterization in a normal bovine cell line. IPA analysis revealed that butyrate exerts a very broad range of effects on many biological pathways through its inhibitory action on HDACs in the MDBK cell line. Our NGS results, with a comparative transcriptomic profiling approach, extended far beyond the findings reported using microarray technologies [10], [20]. The phenomenal number of genes we identified that fall within a broad range of functional categories appear to provide a very detailed molecular basis for the butyrate-induced biological effects.

The stable propagation of genetic information requires that the entire genome of an organism be faithfully replicated only once in each cell cycle. In eukaryotes, this replication is initiated at hundreds to thousands of replication origins distributed over the genome, each of which must be prohibited from re-initiating DNA replication within a single cell cycle [21]. Initiation of DNA replication is a two-step process: First, initiation proteins are assembled onto the replication origin in a stepwise fashion to develop a pre-replication complex. Second, the initiation complex is activated by protein kinases, resulting in the establishment of replication forks. This process is tightly regulated, such that initiation at a given replication origin occurs only once per cell cycle. In addition, initiation is down-regulated in response to agents that damage DNA or block DNA replication.

In eukaryotic cells, cell cycle checkpoint regulation assures the fidelity of cell division. The G1 (first gap phase)/S cell cycle checkpoint controls the passage of eukaryotic cells from the G1 into the S phase. Mitogen-dependent progression through the G1 of the cell-division cycle is accurately regulated to ensure that normal cell division is synchronous with cell growth and that the initiation of DNA synthesis (the S phase) is timed precisely to avoid inappropriate DNA amplification. The G1/S checkpoint control is vital for normal cell division and involves the key components that include cell cycle kinases, CDK4/6-cyclin D and CDK2-cyclin E, and the transcription complex composed of the retinoblastoma protein (Rb) and transcription factor E2F. The activation of E2F is necessary for the G1-S transition. In the present report, CDK4/6 and cyclins E and E2F were significantly down-regulated by butyrate-induced histone acetylation. In contrast, p21, a cell cycle inhibitor protein, was significantly up-regulated. All of these perturbations of gene expression in the G1/S cell cycle checkpoint pathways are consistent with the observed biological effects of butyrate, which induces cell cycle arrest at the G1/S boundary [3].

Butyrate is able to inhibit all class I HDACs. It also seems to affect many other epigenetic-related enzymes by regulating the expression of genes. The missing link is why this inhibition of enzymatic activities, in turn, regulates their own expression at the mRNA level. In this report, we found a vastly complicated depiction of the expression of HDACs induced by butyrate treatment. Whereas the expression of HCACs 7, 8, and 9 are down-regulated, HDACs 5 and 11 are up-regulated, and HDACs 1, 2, 4, and 6 are unchanged (Table S1). HDAC inhibitors that affect the expression of the HDACs themselves have been observed in mouse neural cells [22]. In that report, both TSA and SB indeed elevated the expression of HADC1, HDAC3, HDAC5, and HDAC6, whereas the mRNA levels for HDAC 2 and HDAC7 did not change. The mRNA levels of HDAC8 and HDAC10 were not detectable in these cells. The mechanism and biological relevance of HDAC inhibitors in the regulation of the expression of HDACs is not clear, but may possibly indicate the existence of an auto-regulatory feedback loop for the expression of several HDACs after their activities are inhibited.

Butyrate, as a histone deacetylase inhibitor, can also decrease histone methylation [23], suggesting an interplay between histone acetylation and histone methylation. An emerging possibility is that histone modifications can influence one another. In other words, there may be “crosstalk among histone modification” [24]. Consistently, KDM5B, a specific histone demethylase (H3-trimethyl-K4), was significantly up-regulated by butyrate treatment (Table S1). However, JSRID2, which is directly related to histone methylation and responsible for maintaining the methylation level on histone H3 lysine 27 trimethylation (H3K27me3) [25], was also significantly up-regulated. JARID2 possesses an *in vitro* methyl-protective activity, stabilizing Polycom Repressive Complex 2 (PRC2)-catalyzed H3K27me3 by protecting it from the activity of H3K27 demathylases [26]. These data may indicate that different histone marks (modifications) are differentially regulated and that in turn, differentially regulated histone marks regulate different biological functions [27]. On the other side, a reversal of DNA methylation by butyrate has also recently been reported to occur by the regulation of DNA (cytosine-5-)-methyltransferase 1 (DNMT1) through ERK signaling [28]. In this report, we found that three DNA methyltransferases (DNMTs), DNMT1, DNMT3A, and DNMT3B, were significantly down-regulated by the butyrate treatment (Table S1). While DNMT1 functions in the establishment and regulation of tissue-specific patterns of methylated cytosine residues, DNMT3A and DNMT3B function in the de novo methylation of DNA [29], [30]. These DNMTs are regulated by several mechanisms in terms of their expression and catalytic activity. However, for the first time, our data directly indicated that histone modification has a role in the regulation of the expression of DNMTs, thereby affecting the level of DNA methylation.

The first clear evidence that a six-subunit “origin recognition complex's” (ORC) activity in mammalian cells is regulated by cell cycle–dependent changes in the affinity of the largest subunit (Orc1) for chromatin has been reported [11], [21]. Evidence has since confirmed these findings and extended them to show that mammalian Orc1 is selectively ubiquitinated and phosphorylated during the S-to-M–phase transition, while ORC subunits 2 to 5, which constitute a stable core complex, remain tightly bound to chromatin throughout cell division [31]. In addition, a second mechanism prevents the assembly of a functional ORC until the completion of mitosis: the selective association of Orc1 with Cdk1 (Cdc2)/cyclin A during the G2/M phase of cell division. This association accounted for the appearance in M-phase cells with hyperphosphorylated Orc1 that was subsequently dephosphorylated during the M-to-G1 transition [32]. The rebinding of Orc1 to chromatin follows the same time course as the degradation of cyclin B, suggesting that the exit from mitosis triggers Orc1 binding to chromatin. In fact, the inhibition of Cdk activity in metaphase cells resulted in the rapid binding of Orc1 to chromatin, and NGS profiling shows that all six subunits of ORC are down-regulated by butyrate-induced histone acetylation, adding yet another layer of regulation of ORC activities via the modified expression of those genes. In our previous microarray profiling [10], some of the components of this pathway were found to be perturbed by butyrate-induced gene regulation; however, ORC1 was the only one of the six ORC complex genes that was detected to be a down-regulated gene. In the present report, ORC1 is still the most significantly down-regulated gene, but the other ORC components (ORC2 to ORC6) are all also identified as down-regulated. This result certainly indicates the superb sensitivity of deep RNA sequencing.

We also found significant up-regulation of both BTG1 and BTG2. The BTG family member-2 (BTG2) has antiproliferative activity, and the expression of BTG2 in cycling cells induces the accumulation of hypophosphorylated, growth-inhibitory forms of retinoblastoma protein (Rb) and leads to G1 arrest through the impairment of DNA synthesis. These up-regulated antiproliferation activities are strengthened by the extensive repression of cyclin-dependent kinase and cell cycle-related genes that are clearly associated with the cell growth arrest induced by butyrate.

Tumor protein p53 (TP53, a nuclear protein), transcription factor E2F4, and many other transcription factors, were deregulated by butyrate treatment in the present study. TP53 plays an essential role in the regulation of the cell cycle, specifically in the transition from G0 to G1. It is found in very low levels in normal cells; however, in a variety of transformed cell lines, it is expressed in high amounts and is believed to contribute to transformation and malignancy. P53 is a DNA-binding protein that contains DNA-binding, oligomerization, and transcription activation domains. P53 is postulated to bind as a tetramer to a p53-binding site and activate the expression of downstream genes that inhibit growth and/or invasion, thereby functioning as a tumor suppressor.

P53 has been extensively studied for its function and involvement in butyrate-induced biological effects [33], [34], [35]. Butyrate efficiently suppresses the growth of WT p53-containing cells. It leads to a major G2/M arrest of cells in the presence of p53, while cells without wild-type p53 accumulate mainly in the G1 phase of the cell cycle. Apoptosis induction by butyrate is also greatly reduced in the absence of p53, suggesting that a p53 pathway mediates, in part, growth suppression by butyrate and that p53 status may be an important determinant of chemosensitivity to butyrate [36]. Our data also indicate that the TP53 genes may have different responses and different roles to play in normal and transformed cells. In our dataset, 518 genes were potential targets for TP53 regulation. Among these 518 genes, 238 genes showed expression directions consistent with the activation of TP53. However, one remaining question is why the expression of TP53 was down-regulated, even as its function was more active. As an extremely regulated gene, two major factors may contribute to this complexity of TP53. First, the expression of TP53 is subject to multiple regulations at transcriptional, post-transcriptional, and translational levels, with very complex expression patterns of alternative splicing, alternative promoter usage, and alternative translation. Secondly, the regulation of p53 function is extremely complex and occurs at many levels. Post-translational modifications of p53 (phosphorylation, methylation, acetylation, etc.) alter the functions of p53 (recognition of DNA sequences, interactions with transcription factors at promoters of target genes, etc.) [37]. Indeed, deep RNA-seq and IPA analysis revealed significant changes in the expression of genes related to the molecular function of protein post-translational modification (Figure 2). There are 333 genes related to the phosphorylation of proteins, 80 genes related to the tyrosine phosphorylation of proteins, and 106 genes related to the activation of protein kinase, which is up-regulated by butyrate. The possibility exists that the modification of p53 is affected by butyrate, directly or indirectly. Clearly, more studies are still required to understand the exact roles that TP53 plays in butyrate-induced biological effects.

In conclusion, the acetylation of histone tails is essential for diverse cellular processes, such as DNA replication and cell cycle progression. Butyrate-induced histone hyper-acetylation, however, has some divergent activities, including the induction of cell cycle arrest, gene expression, and apoptosis [3], [10]. The transcriptome characterization of bovine cells using RNAseq identified transcriptional control mechanisms via butyrate. Our results extended our knowledge of the regulatory effects of butyrate on gene expression and will undoubtedly provide insight into the molecular mechanisms of *in vivo* butyrate-induced epigenomic regulation.

## Materials and Methods

### Cell culture and butyrate treatment

Madin-Darby bovine kidney epithelial cells (MDBK, American Type Culture Collection, Manassas, VA., and Catalog No. CCL-22) were cultured in Eagle's minimal essential medium and supplemented with 5% fetal bovine serum (Invitrogen, Carlsbad, CA) in 25 cm^2^ flasks, as described in our previous report [3]. At approximately 50% confluence, during the exponential phase, the cells were treated for 24 hours with 10 mM sodium butyrate (Calbiochem, San Diego, CA). A butyrate concentration of 10 mM was selected as it represents a physiologically relevant dose and has previously been successfully used to evoke desired changes in cell cycle dynamics [3]. Four replicate flasks of cells for both treatment and control groups (i.e., a total of 8 samples) were used for the RNA-seq experiments.

### RNA extraction and sequencing using RNA-seq

Total RNA was extracted using Trizol (Invitrogen, Carlsbad, CA, USA) followed by DNase digestion and Qiagen RNeasy column purification (Qiagen, Valencia, CA, USA), as previously described [5]. The RNA integrity was verified using an Agilent Bioanalyzer 2100 (Agilent, Palo Alto, CA, USA). High-quality RNA (RNA Integrity number or RIN >9.0) was processed using an Illumina TruSeq RNA sample prep kit following the manufacturer's instruction (Illumina, San Diego, CA, USA). After quality control procedures, individual RNA-seq libraries were then pooled based on their respective 6-bp adaptors and sequenced at 50 bp/sequence, read using an Illumina HiSeq 2000 sequencer, as described previously [38]. Approximately 67.5 million reads per sample (mean ± sd  = 67,527,111±8,330,388.6) were generated.

### Data analysis and bioinformatics

Raw sequence reads were first checked using our quality control pipeline. Nucleotides of each raw read were scanned for low quality and trimmed using SolexaQA [39]. Trimmed reads were aligned to the bovine reference genome (Btau 4.0) using TopHat [40]. Each SAM output file per sample from TopHat alignment, along with the GTF file from ENSEMBL bovine genebuild v65.0, were used in the Cuffdiff program in the Cufflink package (v1.3.0) as input files [41] to test for differential expression. Mapped reads were normalized based on the upper-quartile normalization method and Cuffdiff modeled the variance in fragment counts across replicates using the negative binomial distribution as described previously [42].

Differentially-expressed genes in the transcriptome were further analyzed using Gene Ontology (GO) analysis (GOseq) [43]. Enrichment of certain GO terms was determined based on Fisher's exact test. A multiple correction control (permutation to control false discovery rate) was implemented to set up the threshold to obtain the lists of significantly over-represented GO terms.

The molecular processes, molecular functions, and genetic networks following butyrate treatment were further evaluated by analyzing differentially expressed genes using Ingenuity Pathways Analysis (IPA, Ingenuity® Systems, and www.ingenuity.com). IPA is a software application that enables biologists to identify the biological mechanisms, pathways and functions most relevant to their experimental datasets or genes of interest [44], [45], [46], [47], [48].

### Canonical pathway analysis of data sets

Analysis of canonical pathways identified the pathways from the IPA library of canonical pathways that were most significant to the data set. Genes from the data set that were associated with a canonical pathway in the Ingenuity Pathways Knowledge Base were considered for the analysis. The significance of the association between the data set and the canonical pathway was measured in two ways: 1) a ratio of the number of genes from the data set that map to the pathway divided by the total number of genes that map to the canonical pathway was displayed. 2) Fischer's exact test was used to calculate a p-value determining the probability that the association between the genes in the dataset and the canonical pathway was explained by chance alone.

### Functional analysis of data sets

The Functional Analysis identified the biological functions and/or diseases that were most significant to the data set. Genes from the datasets that were associated with biological functions and/or diseases in the Ingenuity Pathways Knowledge Base were considered for the analysis. Fischer's exact test was used to calculate a *p*-value determining the probability that each biological function and/or disease assigned to that data set was due to chance alone.

### Pathways analysis and network generation

A data set containing gene identifiers and corresponding expression values was uploaded into in the application. Each gene identifier was mapped to its corresponding gene object in the Ingenuity Pathways Knowledge Base. These genes, called Focus Genes, were overlaid onto a global molecular network developed from information contained in the Ingenuity Pathways Knowledge Base. Networks of these Focus Genes were then algorithmically generated based on their connectivity.

### Functional analysis of a network

The Functional Analysis of a network identified the biological functions and/or diseases that were most significant to the genes in the network. The network genes associated with biological functions and/or diseases in the Ingenuity Pathways Knowledge Base were considered for the analysis. Fischer's exact test was used to calculate a *p*-value determining the probability that each biological function and/or disease assigned to that network was due to chance alone.

### Network/pathways graphical representation

A network pathway is a graphical representation of the molecular relationships between genes/gene products. Genes or gene products were represented as nodes, and the biological relationship between two nodes were represented as an edge (line). All edges were supported by at least 1 reference from the literature, from a textbook, or from canonical information stored in the Ingenuity Pathways Knowledge Base. The intensity of the node color indicated the degree of up- (red) or down- (green) regulation.

## Supporting Information

Table S1 Transcriptomic analysis of butyrate-induced changes.
(XLSX)

Table S2 Function annotation and comparisons.
(XLSX)

Table S3 Biological Functions induced by butyrate.
(XLSX)

Table S4 List of P53 target genes.
(XLSX)
